# Editorial: Wayfinding and Navigation: Strengths and Weaknesses in Atypical and Clinical Populations

**DOI:** 10.3389/fnhum.2020.588199

**Published:** 2020-10-14

**Authors:** Chiara Meneghetti, Ineke Van Der Ham, Francesca Pazzaglia, Michel Denis

**Affiliations:** ^1^General Psychology Department, University of Padova, Padua, Italy; ^2^Department of Health, Medical and Neuropsychology, Leiden University, Leiden, Netherlands; ^3^Laboratoire d'Informatique pour la Mécanique et les Sciences de l'Ingénieur (LIMSI-CNRS), Orsay, France

**Keywords:** navigation, spatial ability, spatial memory, training, atypical development, Aging, impairments, blind

Navigation is an essential activity of everyday life, related to both work, and leisure. For some populations with certain neurocognitive issues (e.g., those with injuries, genetic syndromes, or other clinical conditions) or characteristics (such as blindness or healthy old age), navigation is fundamental to their autonomy and access to the community. It is a complex activity that entails several stages, from planning a route to reaching a destination (Wiener et al., 2009). The encoding of environmental information in forming a mental representation or cognitive map (Tolman, 1948) and the retrieval and use of that information (Wolbers and Hegarty, 2010) rely on numerous cognitive functions—such as perception, memory, imagination, language, and decision-making—along with social and emotional processes (Dalton et al., 2019).

Our spatial memory of an environment is based on two fundamental frames of reference (Burgess, 2006). One is egocentric and involves mentally arranging the positions of objects in relation to ourselves (subject-to-object). The other is allocentric and establishes relations between objects to determine their respective locations (object-to-object). Navigation is recognized as a large-scale ability supported by small-scale spatial abilities, including the ability to mentally rotate objects or adopt different imaginary views (perspective taking), and processing skills such as visuospatial working memory (VSWM) (Hegarty et al., 2006). Motor abilities are also involved in environment learning (e.g., Voyer and Jansen, 2017). External means (such as navigation aids) can also improve our navigation efficiency. This is a core issue, for instance, in studies on the blind (e.g., Gallay et al., 2013). Brain structures provide the basis for our environment representations and there is neuropsychological evidence indicating that representations with allocentric properties are developed and stored in the medial temporal lobe, parahippocampal gyrus, and hippocampus. The posterior parietal lobe is involved in representations with egocentric properties, and the retrosplenial cortex, in switching between egocentric and allocentric properties of representation. Other brain structures play a part in wayfinding, for instance, the prefrontal cortex supports navigation planning activities (e.g., Lithfous and Després, 2013). The brain regions and networks involved in navigation mechanisms are often examined by considering individuals with brain damage or particular characteristics (e.g., hippocampal volume is smaller in Down syndrome than in matched typically-developing individuals).

With a collection of 15 studies, this special issue advances our knowledge of some navigation and related aspects. Several atypical development (AD) populations are examined in this issue, including: children and adults with William syndrome (WS), who are known to have stronger verbal than spatial abilities (Foti et al.); also comparing them with those with attention deficit hyperactivity disorder [ADHD]; Farran et al.); individuals with Down syndrome (DS; Meneghetti et al.; Himmelberger et al.), known to have stronger spatial than verbal abilities; individuals with autism spectrum disorders (ASD; Cardillo et al.) whose profile in the spatial domain varies. Some contributions examined adults with a cognitive disability (with heterogeneous pathogenesis; Delgrange et al.) who can have difficulty navigating due to their impaired intellectual functioning; and Korsakoff patients, whose memory disorders also affect their recall of spatial information (Janzen et al.).

Other papers examine healthy older adults (Muffato and De Beni) or those with impairments. Elderly people with vascular cognitive impairment (VCI), who have specific egocentric representation difficulties due to their parietal deficit, are compared with cases of early-stage Alzheimer's disease, whose temporal deficit causes specific allocentric representation difficulties (Lowry et al.). These studies mainly examined the allocentric versus egocentric properties of environmental representation using virtual environments (Lowry et al.; Farran et al.; Janzen et al.; Himmelberger et al.), videos of real environments (Muffato and De Beni), or real path learning (Meneghetti et al.). Some studies used spatial tasks to assess navigation-related aspects, such as perspective taking (Cardillo et al.), route planning (Bocchi et al.), and the peripersonal space (Foti et al.), or conducted semi-structured interviews on how respondents solved everyday navigation issues (Delgrange et al.). Two papers focus on individuals with brain lesions: one profiles the navigation-related difficulties of two right-brain-damaged patients using several small- to large-scale tasks (Bocchi et al.); the other concerns an imagery-based rehabilitation program for a patient with right temporal lobe damage suffering from topographical disorientation (Boccia et al.). Two other papers examine blind people and the use of haptic aids, one during navigation (Bharadwaj et al.) and the other for exploring a map before navigating in a real-world setting (Giudice et al.). Finally, two papers assess the malleability of healthy individuals' navigation skills: one involves specific training based on exploration, moving from small areas to larger and more complex environments (McLaren-Gradinaru et al.); the other specific training on the use of egocentric and allocentric navigation strategies (van der Kuil et al.). These findings offer insight into potential rehabilitation programs for individuals with navigation difficulties.

Although different aspects of navigation are examined, each of these studies uses different methods and considers specific populations. The results of these studies indicate that different populations (AD, older adults, Korsakoff patients) can mentally represent spaces and environments with egocentric properties (or sketched; Farran et al.; Meneghetti et al.; Janzen et al.; Muffato and De Beni; Cardillo et al.). Allocentric properties pose more of a challenge, with evidence that allocentric representation is no more impaired in AD than in VCI (Lowry et al.), and can be facilitated by a structured environment (Himmelberger et al.). Mental representations of spaces and environments seem to be supported by general cognitive functioning (as seen in older adults), visuospatial abilities such as mental rotation, VSWM, and the self-reported pleasure people experience when exploring an environment (Cardillo et al.; Foti et al.; Muffato and De Beni; Meneghetti et al.). Motor abilities seem to support spatial performance too, as seen in ASD (Cardillo et al.), but this relation was not found in individuals with WS or ADHD (Farran et al.). In semi-structured interviews, disabled people reported getting lost more frequently in complex environments, and having to ask others for help (Delgrange et al.).

Studies that have examined visual impairment with blind participants have shown the benefits of innovative haptic sources, such as using vibratory signals via a hip-worn belt to navigate, especially in typically noisy everyday environments (Bharadwaj et al.), or presenting vibro-audio maps before navigating (Giudice et al.). A case study contribution explores route planning difficulties in a patient with an occipitoparietal lesion, but not in a patient with a temporoparietal lesion (Bocchi et al.), shedding light on the neurocognitive mechanisms involved in navigation and related aspects of wayfinding. Finally, results obtained with training reveal that navigation skills are malleable, enabling more efficient strategies to be learned such as changing from an egocentric to an allocentric approach (McLaren-Gradinaru et al.; van der Kuil et al.), and enabling even those with topographical disorientation to navigate successfully (Boccia et al.).

To conclude, this special issue expands our understanding of navigation abilities in populations with different characteristics, and how they can be improved by appropriate intervention. Overall, it offers insights that will prompt us to continue to investigate navigation abilities, taking up the challenges faced by different populations and enabling us to create living environments that are more inclusive and accessible.

## Author Contributions

All authors listed have made a substantial, direct and intellectual contribution to the work, and approved it for publication.

## Conflict of Interest

The authors declare that the research was conducted in the absence of any commercial or financial relationships that could be construed as a potential conflict of interest.
